# The effect of different scanning protocols on precision and trueness of intraoral scanning: A pilot trial

**DOI:** 10.4317/jced.62158

**Published:** 2024-10-01

**Authors:** Mustafa Ali Yahya, Mathias Selléus, Deyar Jallal Hadi Deyar Jallal Hadi, Michael Braian, Christel Larsson

**Affiliations:** 1 DDS, MSc. The Institute for Postgraduate Dental Education in Jönköping, Box 1030,551 11 Jönköping, Sweden; 2 DDS, MSc. Oris Dental Sverige AB, S:t Peders Gata 13, 25437 Helsingborg, Sweden; 3 CDT, PhD. Department of Prosthetic Dentistry, Faculty of Odontology, Malmö University, Sweden; 4 CDT, DDS, PhD. Swedish Organisation for Computer Aided Digital Dentistry SWECADD, Baltzarsgatan 25, 211 36 Malmö SWEDEN; 5 DDS, PhD. Associate professor, Faculty of Odontology, Malmö University, Malmö, Sweden and Visiting Associate professor, Faculty of Dentistry, Riga Stradins University, Riga, Latvia

## Abstract

**Background:**

The aim of this study was to investigate how different scanning protocols affect the accuracy (trueness and precision) of intraoral scanning of complete arches with implant cylinders.

**Material and Methods:**

A master model was designed with five cylinders. One scanner, TRIOS2 (3shape), was used to scan the model with four different scan protocols: ROCK (wavelike scanning in a pendulum movement), ZIGZAG (wavelike scanning technique), OBP (occlusal, buccal, and palatal), and OWBP (occlusal, wiggling, buccal, and palatal). A total of 30 scans were performed using each of the four protocols. The master model was digitized with an industrial ISO-certified ATOS scanner. GOM inspect software was used to compare the scans to the master model and evaluate any deviation between the scan protocols and the master model. The data was analyzed using the One Sample t-test (*p*=0,05).

**Results:**

The precision (standard deviation) ranged from 23-83μm for protocol ROCK, 22-147μm for ZIGZAG, 21-170μm for OBP, and 23-116μm for OWBP. The trueness (mean deviation from master model) was 5-41μm for ROCK, 7-97μm for ZIGZAG, -21-29μm for OBP, and 1-24μm for OWBP. All protocols showed statistically significant differences to the master model in multiple distances, except OWBP, which had a single significant difference in comparison to the master model.

**Conclusions:**

Protocol OWBP has a higher trueness than other tested protocols. All tested protocols have higher trueness and precision when scanning smaller distances than inter-arch measurement. Clinical significance;The trueness and precision of intraoral scanning is generally better in smaller spans due to less deviation. The protocol OWBP, that is recommended by the manufacturer, has the least deviating trueness in comparison to the master model.

** Key words:**Accuracy, trueness, precision, intraoral scanner, digital impression, scanning protocol.

## Introduction

The introduction of a digital workflow in dentistry has brought several advantages aimed at correcting some of the main weaknesses with the conventional process. Some examples of these weaknesses are shrinkage of the impression materials and the potential negative effect of disinfectants on the impression material (1-4). Undercuts and angled implants have also been observed to adversely affect conventional impressions due to the force needed when the impression is removed from the mouth (5,6). Several studies show that digital impression (i.e., intraoral 3D scanning) is more time efficient than the conventional impression technique, with a difference of up to 23 minutes faster in a clinical situation (7). In addition, it involves fewer steps where possible errors could occur, which may help prevent a poor fit (4). The goal when producing a prosthetic restoration is to achieve a passive fit. However, it has been shown to be almost impossible to completely avoid a non-passive fit in the manufacturing of implant suprastructures. This may be due to the many steps involved in the prosthetic manufacturing process, such as impressions (8).

ISO 5725-1 uses two terms to describe the accuracy; trueness and precision. Accuracy combines both trueness and precision. It describes the closeness of agreement between the test results and the true value. In a practical sense, it reflects how close the measurement is to the target and how consistent these measurements are (9). The precision refers to the closeness of agreement between independent test results under stipulated conditions. It reflects the reproducibility of the measurement, meaning how consistent the results are when the measurement is repeated under the same conditions, while the trueness refers to the closeness of agreement between the mean of a large number of test results and the true value or the accepted reference value. It reflects how close the measurement is to the actual value (10). To predictably replace conventional impressions with intraoral scanning requires the accuracy to be comparable. A study by Ender *et al*. found the accuracy of conventional impressions to be within +/- 20.4μm (11). A later study showed that using a proper intraoral scanning technique can achieve accuracy that is comparable to conventional impression methods (12). However, some studies show that intraoral scanning is less accurate in comparison to conventional impressions regarding full-arch impressions and impressions for implants (3,11).

Previous studies show that the intraoral scanning technique has a big impact on the restoration misfit (2,12). In addition, recent studies show that the accuracy of intraoral scanning is better when used on a shorter span compared to when scanning a longer span. This could be explained by the stitching effect, where the size of the scanned area contains an accumulation of small inaccuracies, thus resulting in a large error (4,12,13). A scanning protocol that scans the same surface multiple times could potentially reduce the stitching effect and provide improved accuracy.

Therefore, the aim of this study was to investigate how different scanning protocols affect the accuracy (trueness and precision) of intraoral scanning, using one scanning device with associated software. The hypothesis was that a protocol that scans the same surface multiple times will provide the highest accuracy, with minimal deviation from a master model.

## Material and Methods

One scanner was used in this study (TRIOS 2, Pod, v. 1.0.0; 3Shape) with associated software (Software version: 1.4.7.2). A pilot study was performed with four groups of different protocols, same as in the present study, where five scans were performed in each group, with the aim to discover any flaws in the handling and to calibrate the scanner for execution. The pilot study was conducted using a plastic model of a mandible (Black Photoreative Resin for Formlabs 3D printers, Formlabs) with three spheres placed occlusally at the location of the second molar in each quadrant and one lingual of the central incisors. All scans were performed by one operator.

-Master model

**Figure 1:**
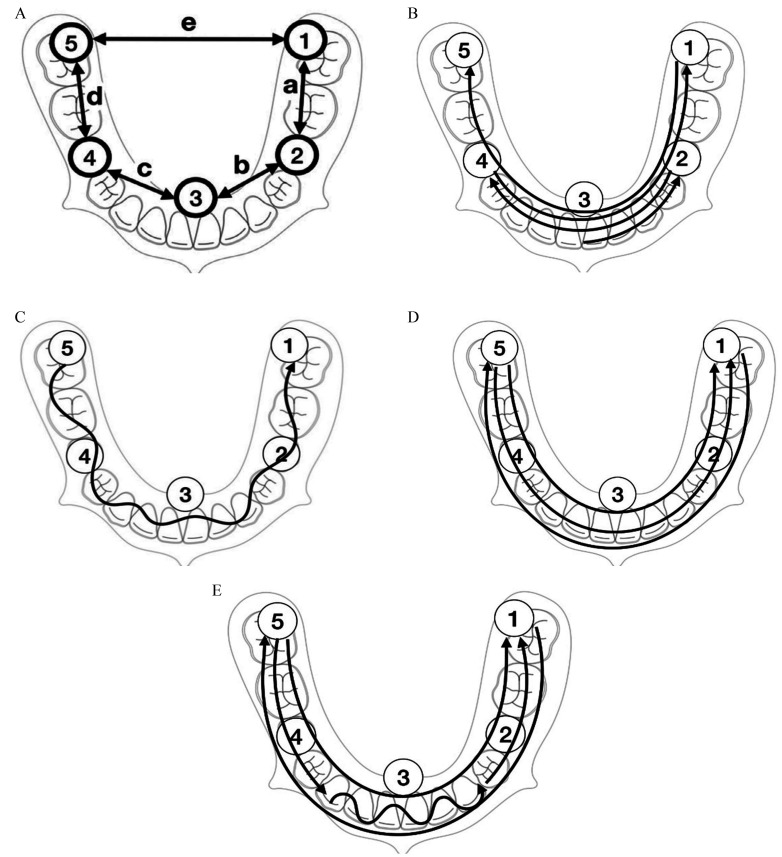
A. Illustration of the master model showing the location of the five different cylinders. The distances between the cylinders were: a=22.567 mm, b=16.544 mm, c=20.552 mm, d=21.544 mm, and e=39.626 mm. B. Protocol ROCK. C. Protocol ZIGZAG. D. Protocol OBP. E. Protocol OWBP.

A master model of a mandible was virtually designed with five cylinders for standardization of the measurement reference. Four cylinders were placed at the occlusal surface at the location of the second premolar and second molar in each quadrant, and one was placed at the lingual surface of the central incisors (Fig. 1A). The model was additively manufactured in Co-Cr (Remanium-Star-CL) using appropriate hardware and software (ConceptLaser M-Lab 100w and 3Shape CAMbridge 2.2.1.3, 2012, CAD program: Magics RP Ver. 13ConceptLaser M-Lab 100w). The surface was then roughened by sandblasting (250 μm AlO2) to create a less reflective surface.

The master model was digitized with an industrially ISO certified ATOS scanner (Scanner: ATOS Capsule Mv70 ScanBox 4105, Program: GOM inspect Professional hotfix6 build 2017 01 13), to which every scan was digitally compared by measuring the fixed central points on the measurement cylinders.

-Protocols

Four different scanning protocols were used.

Protocol ROCK: The scan started lingual of the central incisors at the location of cylinder 3, thereafter the scan continued in a wave like manner from the lingual area to the buccal area. The scanning progressed from the central incisors towards the premolar area in quadrant 3 at the location of cylinder 2, then back and forth in a pendulum movement towards cylinder 4, 1 and then 5 (Fig. 1B). In this protocol, the same surface is scanned multiple times to investigate whether this reduces the stitching effect.

Protocol ZIGZAG: The scans started occlusally of the second molar in quadrant 4 at cylinder 5 and were then performed in a zigzag movement (buccal to lingual), moving towards the second molar in 3rd quadrant at cylinder 1 (Fig. 1C).

Protocol OBP (occlusal, buccal, palatal): The scans started occlusally of the second molar in quadrant 4 at cylinder 5, and then the scan moved towards the second molar in the 3rd quadrant at cylinder 1, only scanning the occlusal area. Thereafter the scan of the buccal area was performed, going from 3rd to 4th quadrant from cylinder 1 to 5. Lastly the lingual area was scanned from 4th to 3rd quadrant form cylinder 5 to 1 (Fig. 1D).

Protocol OWBP (occlusal, wiggling, buccal, palatal): The scans started occlusally of the second molar in quadrant 4 at cylinder 4, and continued until tooth 43, after which the scanning was continued in a wavelike movement (buccal to lingual) until tooth 33. The scan of the occlusal surface resumed towards the second molar in quadrant 3, cylinder 1. Thereafter the scan of the buccal area was performed, going from 3rd to 4th quadrant (cylinder 1 to 5). Lastly the lingual area was scanned from 4th to 3rd quadrant (cylinder 5 to 1) (Fig. 1E).

-Scanning

A total of 30 individual scans were performed per scanning protocol. After each individual scan, the time and required number of images were documented.

A 3D and color calibration of the hardware was performed before the scanning of each individual group according to the manufacturer’s instructions (3shape, 2017-07, Poland, 1KA1731188B).

-Measurement 

GOM inspect (Geomagic Operations Manager inspect; Professional hotfix4 build 2017.0.4.41258, 2017 Germany) was used to perform the measurement on the 120 virtual models, 30 of each protocol, to acquire a distance between the five cylinders (Fig. 1A). The measuring points of the virtual models were placed in the center of the cylinders. The data was analyzed by comparing how much the distances of the cylinders differed from the master model. From that data, a standard deviation and average could be determined for each protocol. The acquired data indicates the trueness of the scanning methods in comparison to the master model.

-Statistical Analysis

The acquired data was analyzed with One Sample t-test to compare each group and measurement individually to the master model (true value). The level of significance was set at alpha = 0.05.

## Results

The time required per scan of the complete arch varied between 1.33 and 4.24 minutes. The minimum number of images for complete-arch scans was 387, while the highest number was 804, as shown in  Table 1. The t-test results showed that every scanning protocol had a statistically significant difference for several of the distances a-e relative to the master model, as shown in  Table 2, except for protocol OWBP, which only had a single significant difference to the master model.

**Figure 2:**
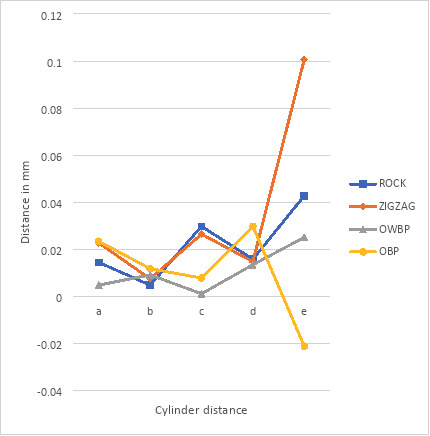
Average deviation from master model (trueness).

**Figure 3:**
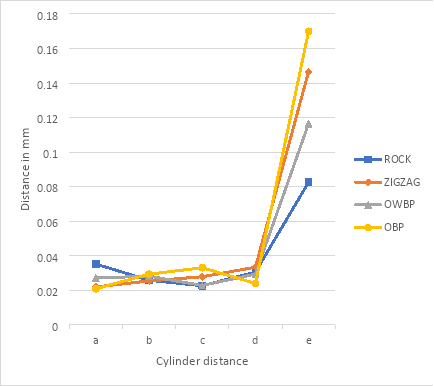
Mean differences and standard deviations between the protocols (precision).

Every group in the study showed an increase in the distance between two fixed points from the master model. The exception was group OBP distance e, where the distance decreased but was not statistically significantly, (Fig. 2). At cylinder distance b, the trueness in all protocols has a generally smaller deviation in comparison to each other from the master model, with a trueness ranging from 0.005 mm to 0.011 mm, as illustrated in Figure 2. Cylinder distance e shows the widest range (0.082 mm to 0.170 mm) and least standard deviation (precision) among the tested protocols. OBP has the largest deviation, while ROCK has the least, as illustrated in Figure 3.

## Discussion

This study investigated how different scanning protocols affected the accuracy (trueness and precision) of intraoral scanning using one single scanning device with associated software. The findings of the present study resulted in the rejection of the hypothesis due to the fact that the protocol with the least deviation of trueness was protocol OWBP, except for the cylinder distance b and e, and this protocol does not scan the same surface as many times as the experimental ROCK protocol.

All protocols showed smaller deviation from the master model in distance b, the smallest distance. This corresponds to findings from previous studies reporting that intraoral scanning is comparable to the conventional technique when working with crowns and smaller restorations up to 4 units (14). However, trueness decreases when scanning longer restorations (14-19) such as distance e in the present study. This suggests that larger prosthetic restorations may have an increased risk of misfit. Similarly, a decrease in the mean distance in relation to the master model, such as observed for the OBP protocol in distance e, may result in a misfit due to a shorter prosthetic restoration. These deviations may be caused by the accumulation of errors that occur during the intraoral scanning process by a stitching effect where the largest distance deviation of scanning of a longer span could be found at one of the endpoints due to the accumulation of errors (2,20-22). However, studies have shown that using a proper intraoral scanning technique can achieve trueness that is comparable to or even better than conventional impression when working with complete-arch restorations (23-30).

There was a wide range in deviation for distance e. The protocols OBP and OWBP had the least deviations of trueness suggesting that these protocols have less accumulation of errors and thus higher trueness. The reason could be other than the way the scans are performed, for example, that the software is adapted to the scanning protocol OWBP and when another scanning technique is used, the software does not function optimally. The same reason explains the outcome for the OBP protocol, as this is similar to OWBP.

On the other hand, the OWBP protocol had a single recorded statistically significant difference at cylinder distance d in comparison to the master model. OBP on the other hand, had a statistically significant difference in three distances a, b, and d in comparison to the master model. This indicates that OWBP has a higher trueness than OBP. This difference may be due to the wiggling that is performed in protocol OWBP, but not in protocol OBP.

In the groups ROCK and ZIGZAG, a statistically significant difference was recorded in four equal distances in comparison to the master model. This indicates that the groups ROCK and ZIGZAG have the same trueness and their trueness is less than the groups OBP and OWBP.

The precision had a larger deviation to the master model than the deviation of the trueness in the same group. In group ROCK, the precision deviated more than the trueness in distance a, b, d and e. In group ZIGZAG, the precision deviated more than the trueness in distance b, c, d and e. In group OBP, the precision deviated more than the trueness in distance b, c and e. Finally, in group OWBP, the precision deviated more than the trueness in distance a, b, c, d and e, indicating a larger deviation of precision than of trueness. This suggests that the lack of precision during the scanning phase could result in a misfit when working with larger restorations, even if the trueness of the machine is acceptable.

Some studies indicate that the tolerance of misfit could be considered as an acceptable fit with a misfit up to 108μm (2,31,32). However, on implants, some studies indicate that a misfit of over 50μm could give problems with the connections to the implants (32). This suggests that suboptimal scanning trueness and precision could be even more problematic when working with implant restorations where a passive fit is argued to be necessary to prevent biological reactions such as marginal bone loss and to prevent screw loosening (2,33). Hussam *et al*. showed that the precision of intraoral scanning was not reliable to scan multiple implants in a completely edentulous arch (22).

The distance referred to as e indicates that longer spans have a deviation that exceeds the maximum limit of 108μm (50μm for implants) in all of the protocols (2,31,32). These results are due to the fact that, even if the trueness is within the set limit, the precision makes the trueness unreliable in longer spans.

## Limitations

Ambient light and temperature are parameters that could impact the accuracy of intraoral scanning (16,34,35). Revilla-León *et al*. showed that the accuracy of intraoral scanning decreases when the temperature is dropped or raised by 5°C, compared to when the scan is performed under the standard temperature of 24 degrees (36). All scans in the present study were performed in the same windowless room and in the same artificial light, i.e. no direct light from windows. Other parameters that can have a significant effect on accuracy are operator experience (6,21,37). One of the authors of this study (M.S) performed all of the scans in the present study; however, the operator had limited experience.

Given that this study did not begin with a null hypothesis, there is a risk of bias towards the tested protocols used in this study; however, the operator performed all of the scans under the same premises where the scans were continued until the program accepted the scan to minimize any errors.

The choice of protocols was based on the fact that these protocols have been used in previous similar studies, except for the protocol ROCK, which was designed for the present study. The master model was updated from spheres to cylinders as a reference point for the measurements spheres were difficult to accurately scan with the chosen protocols.

It is important to note that the findings in this study are based solely on one scanning machine, and all measurements are performed between adjacent cylinders, which means this study cannot make any conclusions about longer spans. To evaluate the fit of longer spans and the effect of different protocols with other hardware, additional studies are necessary.

## Conclusions

Within the limitations of this study, the following conclusions are made:

• Of the tested protocols, the trueness of protocol OWBP had the least statistically significant deviation from the master model.

• For smaller spans (up to a range of approximately three teeth), the trueness and precision was more reliable than inter-arch measurement in all tested protocols.

More studies should be performed to evaluate the effect of different protocols on the trueness and precision when scanning longer spans.

## Figures and Tables

**Table 1:** Number of images (min and max) and time (min and max) required per scan.

Group	Minimum number of images	Maximum number of images	Minimum time per scan (minute)	Maximum time per scan (minute)
ROCK	461	804	2.3	4.24
ZIGZAG	389	783	1.33	3.83
OBP	387	630	1.53	3.00
OWBP	405	717	2.02	3.45

**Table 2:** Mean differences in distance in comparison to the master model. Distances with a statistically significant difference are underlined.

Group	Cylinder distance	Mean (mm)	Master model value (mm)	Standard deviation (precision)	Mean deviation from master model (trueness)	Sig.
ROCK	a	22.56	22.57	0.035	0.014	0.034
	b	16.55	16.55	0.026	0.005	0.331
	c	20.54	20.57	0.023	0.029	0.000
	d	21.53	21.55	0.031	0.015	0.009
	e	39.59	39.63	0.083	0.041	0.008
ZIGZAG	a	22.55	22.57	0.022	0.022	0.000
	b	16.54	16.55	0.025	0.007	0.125
	c	20.54	20.57	0.028	0.025	0.000
	d	21.54	21.55	0.033	0.014	0.023
	e	39.53	39.63	0.147	0.097	0.001
OBP	a	22.55	22.57	0.021	0.023	0.000
	b	16.54	16.55	0.029	0.011	0.038
	c	20.56	20.57	0.033	0.007	0.214
	d	21.52	21.55	0.024	0.029	0.000
	e	39.65	39.63	0.170	-0.021	0.497
OWBP	a	22.57	22.57	0.027	0.005	0.354
	b	16.54	16.55	0.028	0.009	0.091
	c	20.57	20.57	0.023	0.001	0.812
	d	21.54	21.55	0.029	0.013	0.019
	e	39.61	39.63	0.116	0.024	0.249

## Data Availability

The datasets used and/or analyzed during the current study are available from the corresponding author.

## References

[1] Güth JF, Keul C, Stimmelmayr M, Beuer F, Edelhoff D (2013). Accuracy of digital models obtained by direct and indirect data capturing. Clin Oral Investig.

[2] van der Meer WJ, Andriessen FS, Wismeijer D, Ren Y (2012). Application of intra-oral dental scanners in the digital workflow of implantology. PLoS One.

[3] Ajioka H, Kihara H, Odaira C, Kobayashi T, Kondo H (2016). Examination of the Position Accuracy of Implant Abutments Reproduced by Intra-Oral Optical Impression. PLoS One.

[4] Patzelt SB, Emmanouilidi A, Stampf S, Strub JR, Att W (2014). Accuracy of full-arch scans using intraoral scanners. Clin Oral Investig.

[5] Sorrentino R, Gherlone EF, Calesini G, Zarone F (2010). Effect of implant angulation, connection length, and impression material on the dimensional accuracy of implant impressions: an in vitro comparative study. Clin Implant Dent Relat Res.

[6] Giménez B, Özcan M, Martínez-Rus F, Pradíes G (2014). Accuracy of a digital impression system based on parallel confocal laser technology for implants with consideration of operator experience and implant angulation and depth. Int J Oral Maxillofac Implants.

[7] Patzelt SB, Lamprinos C, Stampf S, Att W (2014). The time efficiency of intraoral scanners: an in vitro comparative study. J Am Dent Assoc.

[8] Heckmann SM, Karl M, Wichmann MG, Winter W, Graef F, Taylor TD (2004). Cement fixation and screw retention: parameters of passive fit. An in vitro study of three-unit implant-supported fixed partial dentures. Clin Oral Implants Res.

[9] (2023). Accuracy (trueness and precision) of measurement methods and results - Part 1: General principles and definitions. ISO.

[10] Pesce P, Pera F, Setti P, Menini M (2018). Precision and Accuracy of a Digital Impression Scanner in Full-Arch Implant Rehabilitation. Int J Prosthodont.

[11] Ender A, Mehl A (2013). Accuracy of complete-arch dental impressions: a new method of measuring trueness and precision. J Prosthet Dent.

[12] Ender A, Mehl A (2013). Influence of scanning strategies on the accuracy of digital intraoral scanning systems. Int J Comput Dent.

[13] Luthardt RG, Loos R, Quaas S (2005). Accuracy of intraoral data acquisition in comparison to the conventional impression. Int J Comput Dent.

[14] Abduo J, Elseyoufi M (2018). Accuracy of Intraoral Scanners: A Systematic Review of Influencing Factors. Eur J Prosthodont Restor Dent.

[15] Tan MY, Yee SHX, Wong KM, Tan YH, Tan KBC (2019). Comparison of Three-Dimensional Accuracy of Digital and Conventional Implant Impressions: Effect of Interimplant Distance in an Edentulous Arch. Int J Oral Maxillofac Implants.

[16] Revilla-León M, Subramanian SG, Özcan M, Krishnamurthy VR (2020). Clinical Study of the Influence of Ambient Light Scanning Conditions on the Accuracy (Trueness and Precision) of an Intraoral Scanner. J Prosthodont.

[17] Miyoshi K, Tanaka S, Yokoyama S, Sanda M, Baba K (2020). Effects of different types of intraoral scanners and scanning ranges on the precision of digital implant impressions in edentulous maxilla: An in vitro study. Clin Oral Implants Res.

[18] Ahlholm P, Sipilä K, Vallittu P, Jakonen M, Kotiranta U (2018). Digital Versus Conventional Impressions in Fixed Prosthodontics: A Review. J Prosthodont.

[19] Resende CCD, Barbosa TAQ, Moura GF, Tavares LDN, Rizzante FAP, George FM (2021). Influence of operator experience, scanner type, and scan size on 3D scans. J Prosthet Dent.

[20] Vandeweghe S, Vervack V, Dierens M, De Bruyn H (2017). Accuracy of digital impressions of multiple dental implants: an in vitro study. Clin Oral Implants Res.

[21] Giménez B, Özcan M, Martínez-Rus F, Pradíes G (2015). Accuracy of a digital impression system based on active wavefront sampling technology for implants considering operator experience, implant angulation, and depth. Clin Implant Dent Relat Res.

[22] Mutwalli H, Braian M, Mahmood D, Larsson C (2018). Trueness and Precision of Three-Dimensional Digitizing Intraoral Devices. Int J Dent.

[23] Ribeiro P, Herrero-Climent M, Díaz-Castro C, Ríos-Santos JV, Padrós R, Mur JG (2018). Accuracy of Implant Casts Generated with Conventional and Digital Impressions-An In Vitro Study. Int J Environ Res Public Health.

[24] Papaspyridakos P, Gallucci GO, Chen CJ, Hanssen S, Naert I, Vandenberghe B (2016). Digital versus conventional implant impressions for edentulous patients: accuracy outcomes. Clin Oral Implants Res.

[25] Amin S, Weber HP, Finkelman M, El Rafie K, Kudara Y, Papaspyridakos P (2017). Digital vs. conventional full-arch implant impressions: a comparative study. Clin Oral Implants Res.

[26] Alikhasi M, Siadat H, Nasirpour A, Hasanzade M (2018). Three-Dimensional Accuracy of Digital Impression versus Conventional Method: Effect of Implant Angulation and Connection Type. Int J Dent.

[27] Abdel-Azim T, Zandinejad A, Elathamna E, Lin W, Morton D (2014). The influence of digital fabrication options on the accuracy of dental implant-based single units and complete-arch frameworks. Int J Oral Maxillofac Implants.

[28] Menini M, Setti P, Pera F, Pera P, Pesce P (2018). Accuracy of multi-unit implant impression: traditional techniques versus a digital procedure. Clin Oral Investig.

[29] Pera F, Pesce P, Bagnasco F, Pancini N, Carossa M, Baldelli L (2023). Comparison of Milled Full-Arch Implant-Supported Frameworks Realised with a Full Digital Workflow or from Conventional Impression: A Clinical Study. Materials (Basel).

[30] Marques S, Ribeiro P, Falcão C, Lemos BF, Ríos-Carrasco B, Ríos-Santos JV (2021). Digital Impressions in Implant Dentistry: A Literature Review. Int J Environ Res Public Health.

[31] Ma T, Nicholls JI, Rubenstein JE (1997). Tolerance measurements of various implant components. Int J Oral Maxillofac Implants.

[32] Braian M, De Bruyn H, Fransson H, Christersson C, Wennerberg A (2014). Tolerance measurements on internal- and external-hexagon implants. Int J Oral Maxillofac Implants.

[33] Jemt T, Lindén B, Lekholm U (1992). Failures and complications in 127 consecutively placed fixed partial prostheses supported by Brånemark implants: from prosthetic treatment to first annual checkup. Int J Oral Maxillofac Implants.

[34] Arakida T, Kanazawa M, Iwaki M, Suzuki T, Minakuchi S (2018). Evaluating the influence of ambient light on scanning trueness, precision, and time of intra oral scanner. J Prosthodont Res.

[35] Revilla-León M, Jiang P, Sadeghpour M, Piedra-Cascón W, Zandinejad A, Özcan M (2020). Intraoral digital scans-Part 1: Influence of ambient scanning light conditions on the accuracy (trueness and precision) of different intraoral scanners. J Prosthet Dent.

[36] Revilla-León M, Gohil A, Barmak AB, Gómez-Polo M, Pérez-Barquero JA, Att W (2023). Influence of ambient temperature changes on intraoral scanning accuracy. J Prosthet Dent.

[37] Giménez B, Özcan M, Martínez-Rus F, Pradíes G (2015). Accuracy of a Digital Impression System Based on Active Triangulation Technology With Blue Light for Implants: Effect of Clinically Relevant Parameters. Implant Dent.

